# The complete chloroplast genome sequence of *Platanus × hispanica* from Sichuan province, China, a medicinal plant and phylogenetic analysis

**DOI:** 10.1080/23802359.2021.2002204

**Published:** 2021-11-24

**Authors:** Dan Zhao, Zhu Hou

**Affiliations:** a College of Science and Technology, Xinyang College, Xinyang, Henan, China; b China West Normal University, Nanchong, Sichuan, China

**Keywords:** *Platanus × hispanica*, chloroplast genome, phylogenetic analysis

## Abstract

*Platanus × hispanica* is a tree species with high ornamental value, which complete chloroplast(cp) genome was sequenced, assembled and annotated. The chloroplast genome of *P. hispanica* was 161,717 bp in length, including a large single copy (LSC) region of 92,106, a small single copy (SSC) region of 19,449 bp, and two inverted repeat (IR) regions of 25,081 bp. The GC contents of LSC, SSC, IR, and whole genome are 36.2, 33.0, 43.4, and 38.0%, respectively. There are 131 genes annotated, including 86 protein-coding genes, 37 tRNA genes, and 8 rRNA genes. The phylogenetic analysis revealed that *P. hispanica* was most related to *Platanus occidentalis* as a sister group with 100% bootstrap support. The complete chloroplast genome sequences of *P. hispanica* will provide valuable genomic information to further illuminate phylogenetic classification of *Platanus* genus.

*Platanus × hispanica* Mill. ex Münchh.,1770 belongs to *Platanus* of Platanaceae family (Davis et al. 2002). *Platanus × hispanica* (syn. *Platanus × acerifolia* and *Platanus × hybrida*), the London Plane or Hybrid Plane, is a tree in the genus *Platanus*. (Roberts et al. 2015). *Platanus × hispanica* is a large deciduous tree growing to 20–35 m (exceptionally over 40 m) tall, with a trunk up to 3 m or more in circumference. The bark is usually pale grey-green, smooth and exfoliating, or buff-brown and not exfoliating. The leaves are thick and stiff-textured, broad, palmately lobed, superficially maple-like, the leaf blade 10–20 cm long and 12–25 cm broad, with a petiole 3–10 cm long. (Li et al. 2017). For a better understanding of the relationships of *P. hispanica* and other *Platanus* species, it is necessary to reconstruct a phylogenetic tree based on high-throughput sequencing approaches. In this present study, we reported and characterized the complete chloroplast (cp) genome of *P. hispanica* based on Illumina pair-end sequencing and compared it with other genus cp genome sequences. The result would supply valuable information for the evolution process and conservation genetics of *P. hispanica*.

The sample of *P. hispanica* was collected from Nanchong, Sichuan province, China (106°08′E; 30°79′N). A specimen was deposited at the herbarium of College of Science and Technology, Xinyang College, Xinyang, Henan, China (http://www.nymc.edu.cn/, Zhao Dan, 495685076@qq.com) under the voucher number ZJ001. The DNA sample was properly stored at −80 °C at Key Laboratory of Department of Chinese Medicine, Nanyang, China. Total genomic DNA was extracted using the DNA Secure Plant Kit (Tiangen Biotech, Beijing, China) following the manufacturer’s protocol. Library preparation and genomic sequencing on the Illumina Hiseq 2500 platform were conducted by Benagen (Benagen Inc., Wuhan, China). The raw sequence data has been deposited into NCBI SRA with project accession of SRR14793493. The raw data was filtered using Trimmomatic Version 0.32 with default settings (Bolger et al. 2014). The filtered output was a 5.4 Gb raw data of 150 bp paired-end reads.The obtained paired-end reads were assembled using SPAdes v.3.9.0 (Nurk et al. 2017). The assembled sequence was annotated in MPI-MP CHLOROBOX (https://chlorobox.mpimp-golm.mpg.de/geseq.html) via GeSeq (Tillich et al. 2017) with the reference cp genome of *P. occidentalis* (NC_008335), and then corrected using Geneious Prime v2020.2 (Kearse et al. 2012). Finally, the complete chloroplast genome of *P. hispanica* was submitted to GenBank (Accession No. MZ128519).

The chloroplast genome of *P. hispanica* was 161,717 bp in length, including a large single copy (LSC) region of 92,106, a small single copy (SSC) region of 19,449 bp, and two inverted repeat (IR) regions of 25,081 bp. The GC contents of LSC, SSC, IR, and whole genome are 36.2, 33.0, 43.4, and 38.0%, respectively. There are 131 genes annotated, including 86 protein-coding genes, 37 tRNA genes, and 8 rRNA genes.

To further investigate its taxonomic status, a Maximum-likelihood (ML) tree was constructed based on complete chloroplast genome sequences using MEGA 7.0 (Kumar, Stecher, & Tamura, 2016) with 1000 bootstrap replicates. The program operating parameters were set as follows: a Tamura 3-parameter (T92) nucleotide substitution model with 1000 bootstrap repetitions, accompanied by Gamma distributed with Invariant site (G + I) rates, and partial deletion of gaps/missing data. We used the complete *P. hispanica* cp genome to reconstruct the phylogenetic relationships, 13 other related species were achieved from GenBank, and the species of *Ranzania japonica* and *Berberis oiwakensis* were used as outgroup. Information of the 15 related species is shown in Figure 1. The 16 chloroplast genome sequences were aligned with MAFFT (Katoh and Standley 2013), and then the Maximum-likelihood (ML) tree was constructed. The phylogenetic analysis revealed that *P. hispanica* was most related to *Platanus occidentalis* as a sister group with 100% bootstrap support. The complete chloroplast genome sequences of *P. hispanica* will provide valuable genomic information to further illuminate phylogenetic classification of *Platanus* genus.

**Figure 1.**
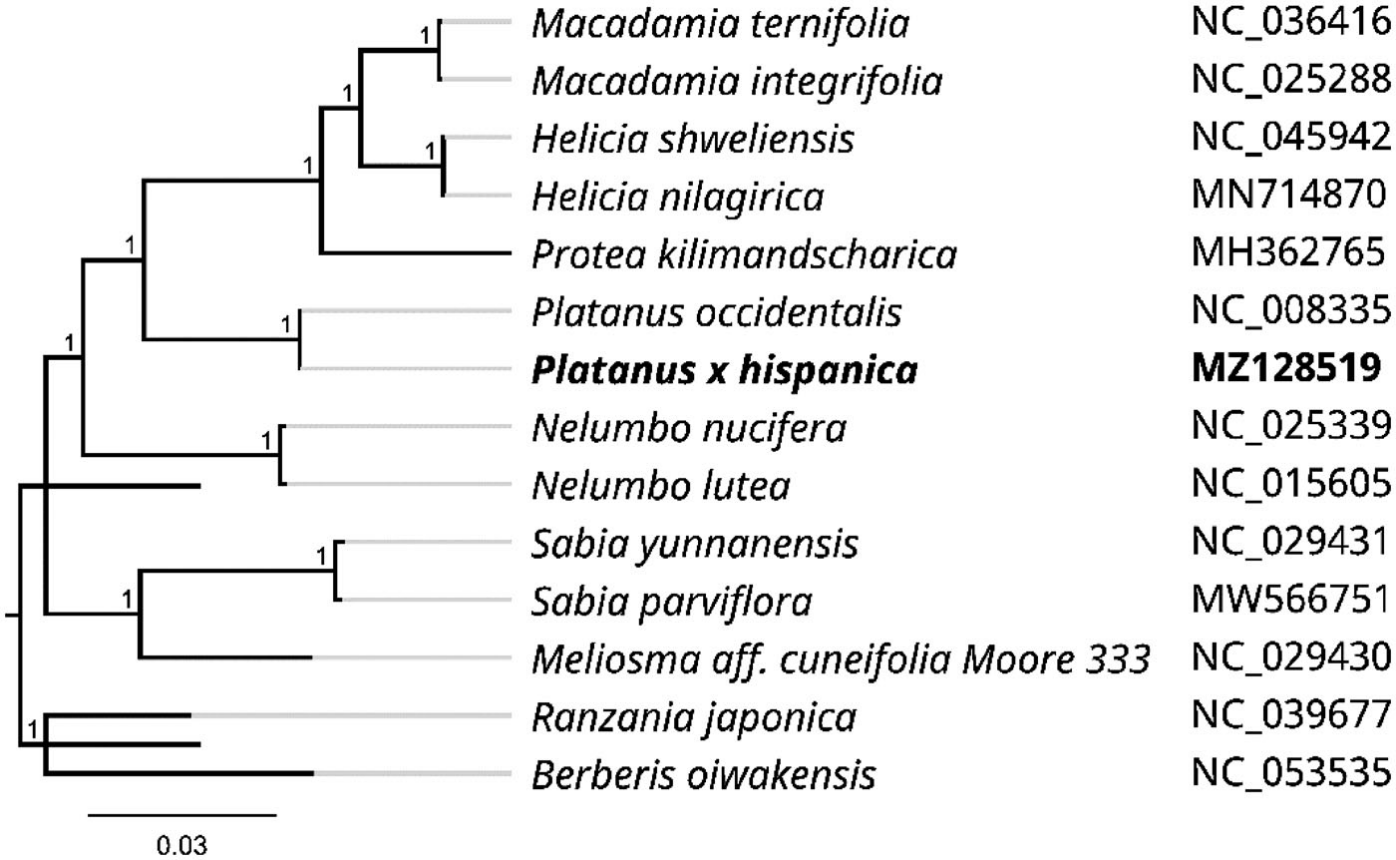
Maximum likelihood phylogenetic tree of *Platanus × hispanica* and other related species based on the complete chloroplast genome sequence.

## Data Availability

The genome sequence data that support the findings of this study are openly available in GenBank of NCBI at (https://www.ncbi.nlm.nih.gov/) under the accession No. MZ128519. The associated BioProject, SRA, and Bio-Sample numbers are PRJNA737038, SRR14793493, and SAMN19678358, respectively.
